# Accurate and Comprehensive Spectrum Characterization for Cavity-Enhanced Electro-Optic Comb Generators

**DOI:** 10.3390/nano12213907

**Published:** 2022-11-05

**Authors:** Ruitao Yang, Jinxuan Wu, Hongxing Yang, Haijin Fu, Liang Yu, Xu Xing, Yisi Dong, Pengcheng Hu, Jiubin Tan

**Affiliations:** 1 Center of Ultra-Precision Optoelectronic Instrument Engineering, Harbin Institute of Technology, Harbin 150080, China; 2 Key Lab of Ultra-Precision Intelligent Instrumentation, Harbin Institute of Technology, Ministry of Industry and Information Technology, Harbin 150080, China

**Keywords:** optical frequency comb, cavity resonators, electrooptic modulation

## Abstract

Cavity-enhanced electro-optic comb generators (CEEOCGs) can provide optical frequency combs with excellent stability and configurability. The existing methods for CEEOCGs spectrum characterization, however, are based on approximations and have suffered from either iterative calculations or limited applicable conditions. In this paper, we show a spectrum characterization method by accumulating the optical electrical field with respect to the count of the round-trip propagation inside of CEEOCGs. The identity transformation and complete analysis of the intracavity phase delay were conducted to eliminate approximations and be applicable to arbitrary conditions, respectively. The calculation efficiency was improved by the noniterative matrix operations. Setting the maximum propagation count as 1000, the spectrum of the center ±300 comb modes can be characterized with merely the truncation error of floating-point numbers within 1.2 s. More importantly, the effects of all CEEOCG parameters were comprehensively characterized for the first time. Accordingly, not only the exact working condition of CEEOCG can be identified for further optimization, but also the power of each comb mode can be predicted accurately and efficiently for applications in optical communications and waveform synthesis.

## 1. Introduction

Optical frequency comb (OFC) is composed of a series of equally spaced and phase coherent frequency components [1,2]. Its unique property in the frequency and time domains brings revolutionary development in the fields of precision spectroscopy [3,4,5], optical communication [6,7], waveform synthesis [8,9,10], and precision metrology [11,12,13] etc. Compared to the OFC generation schemes based on mode-locked lasers [14,15,16] and micro-resonator lasers [17,18], the electro-optic modulators (EOMs)-based OFC generators have some unique advantages [19,20]. OFCs with a high repetition rate up to tens of GHz can be conveniently obtained with a robust and compact setup. Moreover, the central frequency and the repetition rate of the generated OFC can be configured freely and independently [21]. Such advantages make it a perfect multiwavelength laser source for applications in the fields of optical communication and optical arbitrary waveform generation [22].

Limited by the weak EOM interaction strength, the OFCs directly generated by a single EOM suffer from the narrow span of the comb spectrum. The cascade of multiple phase and amplitude EOMs can broaden the OFC spectrum up to tens of comb modes and a few nm of spanning, but no more [23]. Highly nonlinear fiber can be applied to broaden the comb spectrum as well [24]. However, the system structure and size must be increased by adding optical amplification and pulse shaping units to excite the highly nonlinear effect. The shape and phase of the broadened comb spectrum is hard to predict or characterize as well [22].

An alternative wide spectrum EOM comb generation method uses an optical resonator to enhance the modulation process. This method was first proposed by T. Kobayashi et al. in 1972 [25]. Since 1993, a series of great studies on this cavity-enhanced electro-optic comb generator (CEEOCG) have been conducted by M. Kourogi, L. R. Brothers, A. S. Bell, J. Ye, and U. Sterr et al. [26,27,28,29,30,31,32,33,34]. As a new type of comb source, the comb spectrum characterization is the priority. In [26], M. Kourogi proposed the first CEEOCG spectrum characterization method by iteratively accumulating the inter-mode power coupling. The influence of material dispersion was investigated and compensated to achieve a broader spectrum range as well [31,32]. As the number of the comb modes and iterative processes are both theoretically infinite, however, even Kourogi himself admits that this calculation is too complicated [26]. To simplify the process, only the ±15 adjacent comb modes were iteratively calculated with matrix operations [30]. However, the iterative accumulation principle of this spectrum characterization method significantly magnified the errors from the limited comb modes and truncations during signal processing. The comb of a wider spectrum from CEEOCG still cannot be characterized accurately [35,36]. To avoid the complex iterative calculations, Kourogi proposed an exponential approximation method to characterize the spectrum of CEEOCGs [26]. However, the abundant mathematical approximations severely limit the accuracy of the spectrum characterization. Therefore, the existing CEEOCG spectrum characterization methods all suffer from insufficient accuracy due to either the iterative error accumulation or the abundant mathematical approximations.

At the same time, our previous research has proved that the accurate identification of the CEEOCG working condition is required for the fine adjustments of CEEOCG to achieve its optimized performance [37]. However, the CEEOCG comb spectrum has not been comprehensively characterized for the different working conditions of CEEOCG. Consequently, the lack of an accurate and comprehensive spectrum characterization method prohibits the wider application of CEEOCGs.

In this paper, we propose an accurate and comprehensive spectrum characterization method for CEEOCGs by accumulating the optical electrical field with respect to the count of the round-trip propagation inside of the CEEOCG cavity. Different from the existing methods, the proposed method is free from the iterative calculation of the power coupling among the generated comb modes. More importantly, there is no mathematical approximation introduced in the derivation. The influence of the limited count of the round-trip propagation was analyzed in detail and the accuracy of different methods was compared. Based on the proposed method, the influence of all the parameters was investigated independently and jointly. To our knowledge, it is the first comprehensive analysis of CEEOCG based on a highly accurate spectrum characterization method.

## 2. The Existing Methods for the Spectrum Characterization of CEEOCGS

**Figure 1:**
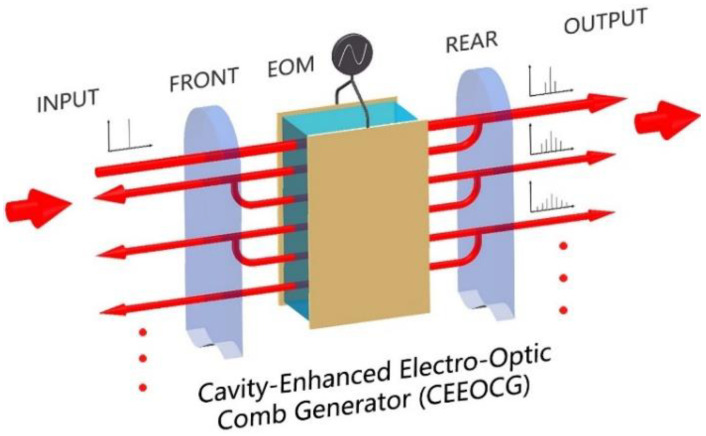
Schematic of the cavity-enhanced electro-optic comb generator (CEEOCG) with a spatial linear cavity structure. EOM: electro-optic modulator.

A CEEOCG consists of an EOM inside a spatial linear cavity or an integrated ring cavity. As these two structures share the same essential principle, we mainly discuss the former type in this paper. A schematic of a typical spatial linear cavity CEEOCG is shown in Figure 1. When a single-wavelength seed laser is incident into the CEEOCG, it oscillates inside the cavity and passes through the EOM multiple times. Driven by a radio-frequency (RF) signal, the EOM introduces phase modulation sidebands to the seed laser. This effect of sideband generation is greatly enhanced by the repetitive beam propagations through the EOM and ensures OFC generation. As the comb modes come from the sidebands of the EOM phase modulation, the repetition rate of the generated comb is determined by the frequency of the EOM modulation signal.

To characterize the comb spectrum, the power coupling effect among the comb modes was analyzed for the first time by M. Kourogi in [26]. As the total spectrum of the generated comb is composed of all the modulation sidebands, the electrical field of the transmission output from CEEOCG can be expressed as:

(1) $$E_{t}=\sum_{k}E_{k}\exp\left[j\left(2\pi v_{0}+k\omega_{m}\right)t\right],$$

where *E_k_* is the electrical field amplitude of the *k*-th order sideband, *ν*_0_ is the frequency of the incident seed laser and *ω_m_* is the angular frequency of the EOM phase modulation. When the beam propagates inside of the linear cavity for a complete round-trip, the power coupling among the sidebands can be expressed as:

(2) $${E^{\prime }}_{k}=t_{1}E_{k\mathrm{in}}+r_{1}r_{2}\exp\left(j2\phi_{k}\right)\sum_{q=-\infty }^{\infty }J_{k-q}\left(2\beta \right)E_{q},$$

where *t*_1_ is the electrical field amplitude transmission coefficient of the input mirror, *E_kin_* is the electrical field of the *k*-th order sideband contained in the input laser spectrum, *r*_1_ and *r*_2_ stand for the electrical field amplitude reflection coefficient of the front input and rear output cavity mirrors, respectively and *ϕ_k_* is the round-trip phase delay of the *k*-th order sideband. For a sideband of the *q*-th order, the power coupling efficiency is described by the first kind Bessel function *J_k−q_*(2*β*) of the (*k*−*q*)-th order, where *β* is the phase modulation index of the EOM. As there are two processes of phase modulation in a round-trip transmission inside of the linear cavity, the modulation index in Equation (2) is doubled as 2*β*. For an integrated ring cavity with a single section of phase modulation, the modulation index should be set as *β* [35,36].

It should be noticed that all the generated sidebands contribute to the *k*-th order sideband after a round-trip propagation. Therefore, the accumulation of all the coupled power from each sideband is required in Equation (2). At the same time, the power of the other sidebands is simultaneously changed together with the *k*-th order. To analyze the generated comb spectrum precisely, therefore, an iteration calculation of the inter-mode power coupling has to be applied. In fact, even the proposer M. Kourogi himself admitted that such an iteration calculation is too complicated [26].

In [30], an approximation calculation was made with less than ±15 adjacent sidebands, instead of all the sidebands ideally. The solution of a 1024 × 1024 sparse matrix equation was applied to describe the CEEOCG output spectrum. However, there are two problems with this solution. On one hand, there are obvious errors between the simulation and the experimental results for the higher order comb modes. On the other hand, the programming and processing of such an algorithm are still very time consuming.

To achieve a rapid characterization of the CEEOCG comb spectrum, M. Kourogi proposed another simplified exponential model in [26]. According to the transmission function of a standard Fabry–Pérot cavity [36,37,38], the electrical field of the transmission beam from a CEEOCG can be expressed as:

(3) Et=Eine−j2πv0t1−Re−jϕF+βsinωmt1−Re−j2ϕF+βsinωmt,

where *R* stands for the equivalent power reflection coefficient of the CEEOCG cavity mirrors. It can be calculated as *R* = *r*_1_ × *r*_2_. The item *β*sin*ω_m_t* represents the phase modulation in a single-pass inside the CEEOCG. *ϕ*_F_ stands for the residual phase delay in a single-pass inside the CEEOCG. After the detailed derivation with a series of mathematical approximations given in the Appendix A, the simplified model can be finally expressed as:

(4) $$I_{tk}={\left|E_{in}\right|}^{2}{\left(\frac{1-R}{2\beta R}\right)}^{2}e^{-\left(\frac{1-R}{\beta R}\right)\left|k\right|}.$$

Nowadays, the comb spectrum model in Equation (4) has become the most popular method for the spectrum characterization of CEEOCGs, including the microring resonator-based CEEOCGs [35,36]. However, the approximations during the derivation of this simplified CEEOCG comb spectrum model cannot be fully met in reality. To compare with our proposed method, we would summarize the important approximation steps as follows.

Firstly, it requires that *ω_m_t* approaches zero to achieve the equivalent infinitesimal replacement of the sine item sin*ω_m_t* as *ω_m_t*. For a commonly used phase modulator of 7~38 mm long and 9~40 GHz modulation frequency [28,39], however, the real value of *ω_m_t* is in the range of 1.87*π* to 2.28*π*. The residual phase will bring obvious error to the model.

More importantly, the derivation of Equation (4) requires the residual phase delay *ϕ*_F_ to approach zero all the time. Only in this case, the exponent item exp[–*j*(*ϕ*_F_+*βω_m_t*)] can be equivalently infinitesimal, replaced as 1–*jβω_m_t*. This requirement severely limits the applicable field of the spectrum model. The real residual phase delay *ϕ*_F_ is effected by the mismatch among the seed laser frequency, phase modulation frequency and the cavity resonance. As another factor that cannot be ignored, the intracavity material dispersion introduces an extra residual phase delay for the higher order comb modes as well.

Therefore, the existing simplified CEEOCG comb spectrum model can only be applied to certain conditions with limited accuracy. Many more cases with parameters of larger range variations cannot be simulated and characterized.

## 3. The Proposed Method for CEEOCG Spectrum Characterization

To derive an accurate and non-iterative method for the CEEOCG comb spectrum characterization, the electrical field of the laser beam was carefully analyzed during its propagation inside the CEEOCG cavity. According to Equation (3), the electrical field of the transmission beam from CEEOCG can be expressed as:

(5) $$E_{t}=E_{in}\left(1-R\right)\sum_{n=0}^{\infty }R^{n}e^{-j\left(2n+1\right)\left(\phi_{F}+\beta \sin\omega_{m}t\right)},$$

where *n* stands for the *n*-th round-trip propagation inside the CEEOCG cavity, i.e., the count of the round-trip propagation. To simplify the exponential term in (5), the Jacobi–Anger identity was applied in the derivation [40]. Accordingly, the electrical field of the transmission beam from CEEOCG can be transformed as:

(6) $$E_{t}=E_{in}\left(1-R\right)\sum_{n=0}^{\infty }R^{n}e^{-j\left(2n+1\right)\phi_{F}}\sum_{k=-\infty }^{\infty }J_{k}\left[\left(2n+1\right)\beta \right]e^{-jk\omega_{m}t},$$

where *J_k_*(*x*) stands for the first kind Bessel function of the *k*-th order. Assuming Equation (6) as a Fourier series, the transmitted electrical field of the *k*-th order sideband can be shown as:

(7) $$\begin{array}{cc} E_{tk} & =E_{in}\left(1-R\right)\sum_{n=0}^{\infty }R^{n}J_{k}\left[\left(2n+1\right)\beta \right]e^{-j\left(2n+1\right)\phi_{F}}\\ & =E_{in}\left(1-R\right)\sum_{n=0}^{\infty }R^{n}J_{k}\left(\beta_{n}\right)e^{-j\phi_{Fn}}, \end{array}$$

where *β_n_* = (2*n* + 1)*β* and *ϕ*_F*n*_ = (2*n* + 1)*ϕ*_F_ stand for the phase modulation index and the residual phase delay of the *n*-th round-trip propagation inside of the CEEOCG cavity. It should be noticed that the transmitted electrical field intensity of the *k*-th order sideband is not related to the power coupling among sidebands in Equation (7). Instead, the irreversible increase of the propagation count n makes Equation (7) a non-iterative equation. To calculate the optical power intensity of the *k*-th order sideband, the electrical field of the *k*-th order sideband is multiplied by its conjugate as:

(8) $$\begin{array}{c} I_{tk}=E_{in}^{2}{\left(1-R\right)}^{2}\left\{J_{k}\left(\beta \right)e^{j\phi_{F}}+RJ_{k}\left(3\beta \right)e^{j3\phi_{F}}\right.\left.+R^{2}J_{k}\left(5\beta \right)e^{j5\phi_{F}}+\cdot \cdot \cdot +R^{n}J_{k}\left(\beta_{n}\right)e^{j\phi_{Fn}}\right\}\\ \;\;\times \left\{J_{k}\left(\beta \right)e^{-j\phi_{F}}+RJ_{k}\left(3\beta \right)e^{-j3\phi_{F}}\right.\left.+R^{2}J_{k}\left(5\beta \right)e^{-j5\phi_{F}}+\cdot \cdot \cdot +R^{n}J_{k}\left(\beta_{n}\right)e^{-j\phi_{Fn}}\right\}. \end{array}$$

The multiplication of the infinite plural terms in (8) can be classified into two categories. The first category contains all the multiplication of the same propagation count *n*. This multiplication process eliminates the exponent terms completely. The result is a summation of *R*^2*n*^*J_k_*^2^(*β_n_*) for *n* from zero to infinity. For the second category, the cross multiplication of different propagation count *n* should be calculated. To simplify the result, the products of the same difference of *n* are gathered. Accordingly, all the imaginary terms of the sine function are cancelled out. The result of cross multiplication for a certain propagation count *n*, named *I_tkn_*_2_, can be expressed as:

(9) $$I_{tkn2}=E_{in}^{2}{\left(1-R\right)}^{2}\left[2R^{n}\cos2n\phi_{F}\sum_{m=0}^{n}R^{2m}J_{k}\left(\beta_{m}\right)J_{k}\left(\beta_{m+n}\right)\right],$$

where *m* stands for the difference of the cross multiplication terms, which can be varied from zero to *n*. When we add up the results from both categories, the power intensity of the *k*-th order sideband can be expressed as:

(10) $$\begin{array}{cc} I_{tk} & =E_{in}^{2}{\left(1-R\right)}^{2}\left\{J_{k}^{2}\left(\beta \right)+R^{2}J_{k}^{2}\left(3\beta \right)\right.+R^{4}J_{k}^{2}\left(5\beta \right)+\cdot \cdot \cdot +R^{2n}J_{k}^{2}\left(\beta_{n}\right)\\ & \;+\;2R\cos2\phi_{F}\left[J_{k}\left(\beta \right)J_{k}\left(3\beta \right)\right.+R^{2}J_{k}\left(3\beta \right)J_{k}\left(5\beta \right)\left.+\cdot \cdot \cdot +R^{2n}J_{k}\left(\beta_{n}\right)J_{k}\left(\beta_{n+1}\right)\right]\\ & \;+\;2R^{2}\cos4\phi_{F}\left[J_{k}\left(\beta \right)J_{k}\left(5\beta \right)+R^{2}J_{k}\left(3\beta \right)J_{k}\left(7\beta \right)\right.\left.+\cdot \cdot \cdot +R^{2n}J_{k}\left(\beta_{n}\right)J_{k}\left(\beta_{n+2}\right)\right]\\ & \;+\;\cdot \cdot \cdot \\ & \;+\;2R^{n}\cos2n\phi_{F}\left[J_{k}\left(\beta \right)J_{k}\left(\beta_{n}\right)+R^{2}J_{k}\left(3\beta \right)J_{k}\left(\beta_{n+1}\right)\right.\left.\left.+\cdot \cdot \cdot +R^{2n}J_{k}\left(\beta_{n}\right)J_{k}\left(\beta_{2n}\right)\right]\right\} \end{array}$$

Inspired by the definition of the Matrix multiplication and Hadamard product of matrices, Equation (10) can be further simplified for computer simulation as follows:

(11) $$\begin{array}{cc} I_{tk}=E_{in}^{2}{\left(1-R\right)}^{2} & \left[\left(A_{Rn}\circ C_{Jk\left(0\sim n\right)}\right)C_{Jk\left(0\sim n\right)}^{T}\right.\\ & \;+\;2R\cos2\phi_{F}\left(A_{Rn}\circ C_{Jk\left(0\sim n\right)}\right)C_{Jk\left(1\sim n+1\right)}^{T}\\ & \;+\;2R^{2}\cos4\phi_{F}\left(A_{Rn}\circ C_{Jk\left(0\sim n\right)}\right)C_{Jk\left(2\sim n+2\right)}^{T}\\ & \left.\;+\;\cdot \cdot \cdot +2R^{n}\cos2n\phi_{F}\left(A_{Rn}\circ C_{Jk\left(0\sim n\right)}\right)C_{Jk\left(n\sim 2n\right)}^{T}\right], \end{array}$$

(12) $$A_{Rn}=\left[1\;R^{2}\;\cdot \cdot \cdot \;R^{2n}\right],$$

(13) $$C_{Jk\left(0\sim n\right)}=\left[J_{k}\left(\beta \right)\;J_{k}\left(3\beta \right)\;\cdot \cdot \cdot \;J_{k}\left(\beta_{n}\right)\right],$$

(14) $$C_{Jk\left(1\sim n+1\right)}^{T}={\left[J_{k}\left(3\beta \right)\;J_{k}\left(5\beta \right)\;\cdot \cdot \cdot \;J_{k}\left(\beta_{n+1}\right)\right]}^{T},$$

(15) $$C_{Jk\left(2\sim n+2\right)}^{T}={\left[J_{k}\left(5\beta \right)\;J_{k}\left(7\beta \right)\;\cdot \cdot \cdot \;J_{k}\left(\beta_{n+2}\right)\right]}^{T},$$

(16) $$C_{Jk\left(n\sim 2n\right)}^{T}={\left[J_{k}\left(\beta_{n}\right)\;J_{k}\left(\beta_{n+1}\right)\;\cdot \cdot \cdot \;J_{k}\left(\beta_{2n}\right)\right]}^{T},$$

where (*A_Rn_* ○ *C_Jk_*_(0~*n*)_) stands for the Hadamard product of the *A_Rn_* and *C_Jk_*_(0~*n*)_ vectors, which multiplies the two vectors element by element. The upper corner mark T is the symbol of vector transposition.

As the residual phase delay of a single-pass inside of the CEEOCG, *ϕ*_F_ varies with the working condition of CEEOCG in real time. It consists of the mismatch phase delay *ϕ*_α_ between the seed laser frequency and cavity resonance, the mismatch phase delay *ϕ*_Δ*f*_ between the cavity resonance and phase modulation, and the phase delay *ϕ*_D_ from the intracavity material dispersion. Ignoring the phase delay of 2*nπ* from even times of interface reflections, the residual phase delay of single-pass *ϕ*_F_ can be expressed as:

(17) $$\begin{array}{cc} \phi_{F} & =\phi_{\alpha }+\phi_{\Delta f}+\phi_{D}\\ & =\frac{\pi \delta v}{v_{\mathrm{FSR}}}+\frac{k\pi \Delta f_{m}}{v_{\mathrm{FSR}}}+\frac{\mathrm{GVD}L_{c}{\left(2\pi kf_{m}\right)}^{2}}{2}, \end{array}$$

where *ν*_FSR_ is the free spectral range (FSR) of the CEEOCG cavity, *δν* is the frequency difference between the seed laser and the adjacent cavity resonance, *f_m_* stands for the frequency of phase modulation, *ω_m_* = 2*πf_m_*. Δ*f_m_* is the mismatch between the phase modulation frequency and the cavity FSR, and GVD and *L_c_* stand for the group velocity dispersion and the length of the EOM crystal. According to Equation (17), the phase delay *ϕ*_α_, *ϕ*_Δ*f*_ and *ϕ*_D_ are all non-zero frequency dependent parameters in practical applications.

The complete proposed method for CEEOCG spectrum characterization is composed of Equations (11)–(17). It should be noted that there is no more iterative calculation in this method. Once the parameters are determined, the comb spectrum of the CEEOCG can be calculated with a straight process. To characterize the CEEOCG comb spectrum with higher time efficiency, the vectors of *A_Rn_* and *C_Jk_*_(0~2*n*)_ can be generated with the preset parameters and stored in advance. In this case, the calculation of (11)–(17) requires the summation of matrix multiplication and element-by-element multiplication only. All the processes can be easily and rapidly realized by the existing matrix computation software.

More importantly, it should be noted that there is no more mathematical approximation during the above derivation process. The proposed method for CEEOCG comb spectrum characterization is based on the accumulation of the Bessel function results with the phase modulation index *β_n_* of the round-trip propagation count *n*. Thus, the simulation accuracy of the proposed method is, in principle, determined by the maximum round-trip propagation count *n*_max_ only. However, it should be noted that the truncation error of the floating-point numbers will influence the simulation accuracy as well, even for our non-iterative method. According to the ISO/IEC international standard 60559-2020 for floating-point arithmetic (i.e., IEEE standard 754-2019) [41], the minimum distance between two adjacent double-precision numbers is 2^−52^, i.e., approximately 2.220446 × 10^−16^. For the quadruple and even octuple precision floating point numbers, the truncation error is down to 2^−112^ and 2^−237^, respectively. For the newly accumulated term Δ*I_tk_* of (10) with the increasing of the round-trip propagation count from *n*_max_ to *n*_max_ + 1, the attenuation property of the Bessel function ensures the decrease of Δ*I_tk_* to below this truncation error when *n*_max_ is large enough. Hence, the convergence of the proposed method can be proved as well.

**Figure 2:**
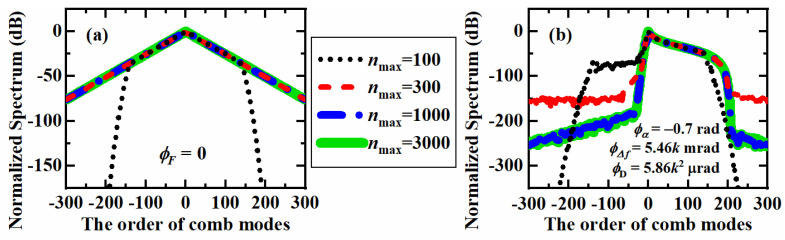
Simulated comb spectrums of the center ±300 combs with different maximum round-trip propagation count *n*_max_ of 100, 300, 1000 and 3000. (**a**) Simulation with residual phase delay *ϕ*_F_ = 0, (**b**) Simulation with *ϕ*_F_ = −0.7 + 5.46 × 10^−3^k + 5.86 × 10^−6^k^2^ rad, corresponding to the mismatch phase delay *ϕ*_α_ = −0.7 rad, the mismatch frequency Δ*f*_m_ = 2 MHz, the cavity FSR *ν*_FSR_ = 9.2 GHz, the modulation frequency *f*_m_ = 9.2 GHz, the GVD and length of LiNbO3 EOM being 350.74 fs^2^/mm and 10 mm, respectively.

To analyze the influence of *n*_max_ on the accuracy of the proposed method quantitatively, the spectrum of the center ±300 modes was simulated with *n*_max_ = 100, 300, 1000 and 3000. The power reflection coefficient *R* and phase modulation index *β* were set to be 96% and 0.7 rad, respectively. When the residual phase delay *ϕ*_F_ = 0, the simulated spectrum is shown in Figure 2a, where the simulated curves of *n*_max_ = 300, 1000 and 3000 seem to be overlapped with each other. To ensure that *n*_max_ is applicable for a more general case of non-zero *ϕ*_F_, the mismatch phase delay *ϕ*_α_ was set to its lower limit of −0.7 rad. The mismatch frequency Δ*f_m_* and cavity FSR *ν*_FSR_ were assumed to be 2 MHz and 9.2 GHz, respectively. Considering the GVD and length of LiNbO_3_ EOM as 350.74 fs^2^/mm [42] and 10 mm, the spectrum simulation is shown in Figure 2b with the modulation frequency *f*_m_ of 9.2 GHz. In this case, the curve of *n*_max_ = 300 separates with the curves of *n*_max_ = 1000 and 3000. Therefore, *n*_max_ =1000 is large enough to simulate the center ±300 combs of a fixed CEEOCG in the working conditions above. To prevent redundant computations without improving the simulation accuracy, *n*_max_ was set as 1000 for the following simulations of the CEEOCG spectrum with the power reflection coefficient *R* and phase modulation index *β* being 96% and 0.7 rad, respectively.

**Figure 3:**
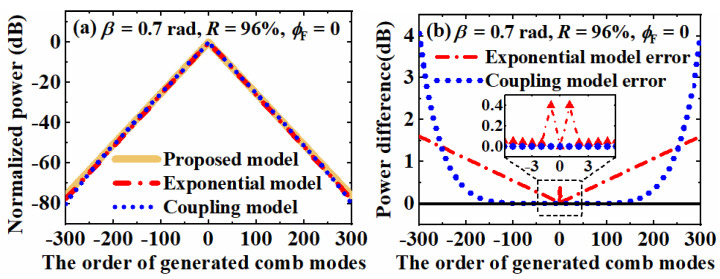
(**a**) Simulated comb spectrums of the center ±300 combs with different models and (**b**) The deviations of the simulated spectrums with the proposed model as reference.

To compare the existing approximation models based on the exponential function and the power coupling among ±15 adjacent sidebands with the proposed model, simulated spectrums and their deviations with the same parameters above are shown in Figure 3a,b, respectively. For the center ±300 modes, the overall comb spectrums of all three models in Figure 3a show an approximately linear power decay with the increasing of comb mode *k*. To compare the efficiency, the simulation time of the proposed model and the power coupling model were characterized by the matrix computation software as 1.2 s and 72.9 s, respectively. The detailed deviations are shown in Figure 3b with the proposed model as a reference. For the exponential approximation model, a linear error up to 1.6 dB can be observed for the higher order comb modes. Meanwhile, there is an extra 0.4 dB error for the ±1st comb modes. In contrast, the error of the power coupling model is nearly zero for the comb modes within the ±100-th order. With the further increasing of comb mode *k*, however, there is a rapid nonlinear raising of deviation up to 4 dB. We attribute this nonlinear error to the incomplete analysis of the power coupling model and the iterated accumulation of the floating-point truncation error. In summary, the exponential model shows a linear error but can only be applied when *ϕ*_F_ = 0. The power coupling model can be applied with arbitrary *ϕ*_F_ value but is not suitable for the analysis of CEEOCGs with abundant comb modes.

## 4. Comprehensive Spectrum Characterization of CEEOCG with Different Parameters

Based on the proposed CEEOCG comb spectrum model above, the influence from the parameters of CEEOCG can be thoroughly characterized, e.g., the power reflection coefficient of cavity mirror *R*, phase modulation index *β* and phase delay *ϕ*_F_. As the phase delay *ϕ*_F_ consists of the dispersion phase delay *ϕ*_D_ and the mismatch phase delay *ϕ*_α_ and *ϕ*_Δ*f*_, their independent and combined impact on the CEEOCG comb spectrum will be revealed in this section.

### 4.1. The Influence of Cavity Mirror Power Reflection Efficiency R and Phase Modulation Index β

**Figure 4:**
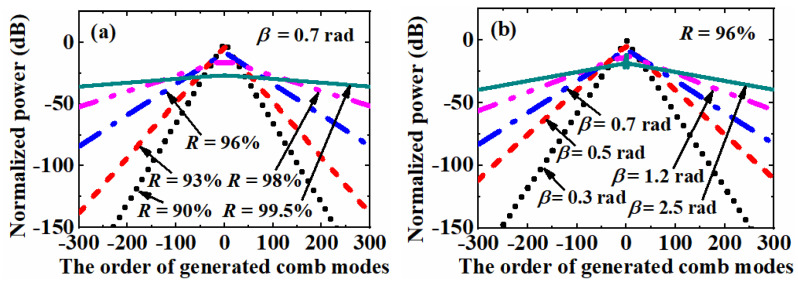
(**a**) Simulated comb spectrum of the center ±300 combs with different power reflection coefficient *R* and (**b**) different phase modulation index *β*. The maximum propagation count *n*_max_ and phase delay *ϕ*_F_ are assumed to be 1000 and 0, respectively.

As shown in Figure 4a, the power distribution of the generated comb spectrum was simulated with a power reflection efficiency *R* of 90%, 93%, 96%, 99% and 99.5%, respectively. In this simulation, the phase modulation index *β* and phase delay *ϕ*_F_ are assumed to be 0.7 rad and 0, respectively. According to the discussion above, the maximum propagation count *n*_max_ was set to be 1000 to simulate the center ±300 comb modes. With the increase of the power reflection efficiency *R* from 90% to 99.5%, the slope of power decay decreased from 0.653 to 0.006 dB per comb mode. Accordingly, the simulation curve became flatter. This simulation result fits the principle of the CEEOCG very well. The increasing of *R* introduces more power oscillation inside the cavity. Consequently, more power is distributed from the center to the higher order modes with the enhanced phase modulation.

When the generated comb spectrum was simulated with phase modulation index *β* of 0.3 rad, 0.5 rad, 0.7 rad, 1.2 rad and 2.5 rad, similar curves are shown in Figure 4b. In this simulation, the power reflection efficiency *R* and phase delay *ϕ*_F_ were assumed to be 96% and 0, respectively. The increasing of phase modulation index *β* from 0.3 rad to 2.5 rad leads to the decreasing of the power decay slope from 0.591 to 0.093 dB per comb mode. It corresponds to a flattening of the simulation curve and a broadening of the spectrum bandwidth. This phenomenon can be explained with an enhanced sideband generation capability during a single pass through the phase modulator. For the simulations with a much lower value of *R* or *β*, the sideband generation effect in a round-trip propagation through the phase modulator is much weaker. Thus, a larger propagation count *n*_max_ is required to simulate the accurate power of higher order comb modes.

### 4.2. The Independent Influence of the Mismatch Phase Delay ϕ_α_, Mismatch Phase Delay ϕ_Δf_ and Dispersion Phase Delay ϕ_D_

**Figure 5:**
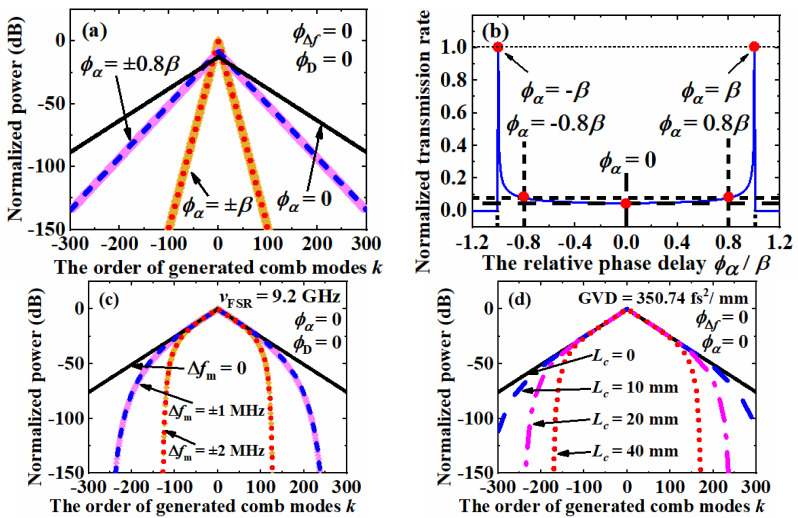
(**a**) Simulated comb spectrum of the center ±300 combs with different mismatch phase delay *ϕ*_α_, (**b**) the normalized transmission rate of the CEEOCG with different mismatch phase delay *ϕ*_α_, (**c**) simulated comb spectrum of the center ±300 combs with different mismatch phase delay *ϕ*_Δ*f*_ and (**d**) different dispersion phase delay *ϕ*_D_.

To reveal the influence of the mismatch phase delay *ϕ*_α_, mismatch phase delay *ϕ*_Δ*f*_ and dispersion phase delay *ϕ*_D_ independently, three simulations were performed with the same power reflection efficiency *R* of 96%, phase modulation index *β* of 0.7 rad and maximum propagation count *n*_max_ of 1000. Assuming the mismatch phase delay *ϕ*_Δ*f*_ and dispersion phase delay *ϕ*_D_ to be zero, the CEEOCG comb spectrums with mismatch phase delay *ϕ*_α_ of 0, ±0.8*β* and ±*β* are shown in Figure 5a. In this situation, the symmetrical curves for different *β* of the same absolute value but opposite sign overlap with each other. With the increasing comb mode order, a linear power decay in the log scale can be observed. The linear slopes are 0.193, 0.421 and 0.806 dB per comb mode for *ϕ*_α_ = 0, ±0.8*β* and ±*β*, respectively. The increasing of the mismatch phase delay *ϕ*_α_ causes a concentration of the optical power to the center combs and there is a significate decrease in the CEEOCG comb bandwidth. This simulation result fits well with the previous reports in [33,34]. As shown in Figure 5b, the transmission rate of the CEEOCG varies with the mismatch phase delay *ϕ*_α_ according to the theoretical analysis in [33,34]. The operation point of *ϕ*_α_ = 0 enables a maximum power coupling from the incident laser mode to the higher order comb modes, which causes a minimum total transmission power. On the contrary, the operation point of *ϕ*_α_ = ±*β* keeps most of the power in the lower order comb modes and achieves the maximum transmission power. 

In Figure 5c, the influence of the mismatch phase delay *ϕ*_Δ*f*_ is simulated by assuming that the mismatch phase delay *ϕ*_α_ and dispersion phase delay *ϕ*_D_ are zero. According to Equation (17), the mismatch phase delay *ϕ*_Δ*f*_ is determined by the order of comb mode *k*, the mismatch frequency between cavity resonance and modulation frequency Δ*f_m_* and the FSR of CEEOCG cavity *ν*_FSR_. When the FSR of CEEOCG cavity *ν*_FSR_ is assumed to be 9.2 GHz, the influence of the mismatch phase delay *ϕ*_Δ*f*_ is simulated with a mismatch frequency Δ*f_m_* of 0, ±1 MHz and ±2 MHz. As the phase delay *ϕ*_Δ*f*_ is proportional to the order of comb mode *k*, the decay of power is accelerated with the increasing of the comb mode order. The total power inside of the CEEOCG cavity does not vary with the mismatch phase delay *ϕ*_Δ*f*_. Therefore, the curves for the comb modes within ±50 orders are overlapped in Figure 5c. With a zero dispersion phase delay *ϕ*_D_, the symmetrical curves of the same absolute value of *ϕ*_Δ*f*_ are consistent with each other as well.

In Figure 5d, the CEEOCG comb spectrums with different values of dispersion phase delay *ϕ*_D_ are simulated by assuming the mismatch phase delay *ϕ*_α_ and *ϕ*_Δ*f*_ are zero. According to Equation (17), the dispersion phase delay *ϕ*_D_ is mainly caused by the phase modulator. When the wavelength of the incident laser is 826.2 nm, the GVD value of the LiNbO_3_ material can be found as 350.74 fs^2^/mm from [39]. Assuming the lengths of EOM crystal *L_c_* to be 0, 10 mm, 20 mm and 40 mm, the comb spectrum is simulated and shown as the black solid line, the blue dash line, the magenta dash-dot line and the red dot line in Figure 5d, respectively. The mode spacing is assumed to be 9.2 GHz. The dispersion phase delay *ϕ*_D_ is proportional to the square of the comb mode order *k*. Accordingly, the accelerated slope of power decay for the curves increases with the mismatch phase delay *ϕ*_D,_ as shown in Figure 5d. As the change of dispersion phase delay *ϕ*_D_ will not influence the total power inside of the CEEOCG cavity, the spectrum overlap of the comb modes within ±100 orders can be explained.

From the analysis above, it is clear that the non-zero mismatch phase delay *ϕ*_α_, *ϕ*_Δ*f*_ and dispersion phase delay *ϕ*_D_ cause anarrowing of the comb spectrum. However, the corresponding comb spectrums are still symmetrical under the independent influence of all three phase delays. With the increasing of comb mode order *k*, only the slope of power decay for the mismatch phase delay *ϕ*_α_ is linear in the dB scale. For both the mismatch phase delay *ϕ*_Δ*f*_ and dispersion phase delay *ϕ*_D_, the power decay is accelerated. Meanwhile, only the mismatch phase delay *ϕ*_α_ will change the total transmission rate of the CEEOCG. For lower order comb modes, the variations of the mismatch phase delay *ϕ*_Δ*f*_ and dispersion phase delay *ϕ*_D_ are not notable.

### 4.3. The Influence of the Mismatch Phase Delay ϕ_α_ and ϕ_Δf_ with a Constant Dispersion Phase Delay ϕ_D_

The analysis above focuses on the independent influence of each phase delay. In a practical application of the CEEOCG, however, the dispersion phase delay *ϕ*_D_ is usually a constant non-zero value. In contrast, the phase delays *ϕ*_α_ and *ϕ*_Δ*f*_ are always variable owing to the mismatch among the incident laser frequency, the cavity resonance frequency and the phase modulation frequency. Thus, the investigation of the influence of the mismatch phase delays *ϕ*_α_ and *ϕ*_Δ*f*_ with a constant value of dispersion phase delay *ϕ*_D_ is of significance. The following spectrum characterizations are made with the same power reflection efficiency *R* of 96%, phase modulation index *β* of 0.7 rad and maximum propagation count *n*_max_ of 1000.

**Figure 6:**
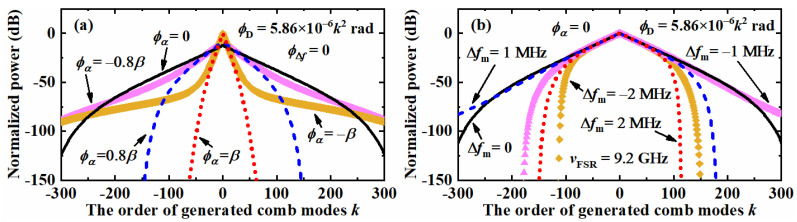
Simulated comb spectrum of the center ±300 combs with a constant dispersion phase delay *ϕ*_D_ of 5.86 × 10^−6^*k*^2^ rad (**a**) different mismatch phase delay *ϕ*_α_ of 0, ±0.8*β* and ±*β*. (**b**) different mismatch phase delay *ϕ*_Δ*f*_. of 0, ±0.8*β* and ±*β*MHz.

Assuming the applied EOM is a 10 mm LiNbO_3_ crystal with a GVD of 350.74 fs^2^/mm [39], the dispersion phase delay *ϕ*_D_ can be calculated with Equation (17) as 5.86 × 10^−6^*k*^2^ rad when the phase modulation frequency is 9.2 GHz. Assuming the mismatch phase delay *ϕ*_Δ*f*_ is zero, the comb spectrums of the mismatch phase delay *ϕ*_α_ = 0, ±0.8*β* and ±*β* were simulated and are shown in Figure 6a. If we compare it to Figure 5a, there are two significant differences. Firstly, the curves of the same absolute value of mismatch phase delay *ϕ*_α_ are separated with different shapes. If we take the cases of *ϕ*_α_ = ±*β* as examples, there is only a slight difference that can be observed for the center comb modes within the ±30 order. With the increase of the mode order *k*, the spectrum decays rapidly for *ϕ*_α_ = *β*. For the phase delay *ϕ*_α_ of −*β*, however, a spectrum broadening effect is shown for the combs from ±50 up to over ±300 orders. The newly generated comb modes exist as two low-energy wings of the spectrum. Thus, this phenomenon could be applied for the broadening of the CEEOCG spectrum with nearly no extra cost. The same situation could be found for the cases of *ϕ*_α_ = ±0.8*β* as well. Secondly, the power decay of the curves is no more linear in the dB scale. The reason is that the introduction of the dispersion phase delay relates the total phase delay to the order of the comb mode. Of course, the symmetry of the curves in Figure 6a is kept the same as in Figure 5a. Besides, the concentration of optical power to lower order comb modes can be found for a larger absolute value of phase delay *ϕ*_α_ as well.

As shown in Figure 6b, the influence of the mismatch phase delay *ϕ*_Δ*f*_ with the same dispersion phase delay *ϕ*_D_ and zero phase delay *ϕ*_α_ was simulated. In these cases, the difference between Figure 5c and Figure 6b can be summarized into two respects. Firstly, the symmetry of the curves themselves is broken by the mismatch phase delay *ϕ*_Δ*f*_ with the help of the dispersion phase delay. The positive mismatch of Δ*f_m_* leads to a left shifting of the simulation curve and vice versa, e.g., the blue dash line and the magenta triangle line in Figure 6b correspond to Δ*f_m_* = 1 MHz and −1 MHz, respectively. The reason for this phenomenon is that the existence of the dispersion phase delay compensates for the positive mismatch of Δ*f_m_* for the negative order of comb modes. At the same time, this effect makes the power of positive order modes decay faster. Of course, a constant dispersion phase delay can only compensate for the mismatch phase delay *ϕ*_Δ*f*_ to a limited extent. Thus, the power decreasing of the negative order modes for the curve of Δ*f_m_* = 2 MHz is no more linear as in the case of Δ*f_m_* = 1 MHz, although the power decreasing rate of the negative order modes is always lower than the positive order modes. Secondly, the simulation curves of the same absolute value but opposite sign are symmetrical to each other. This phenomenon further proves that the change of curve shape from Figure 5c to Figure 6b is caused by a constant dispersion phase delay. At the same time, a reverse of the above phenomenon can be expected with an EOM crystal of negative GVD value.

**Figure 7:**
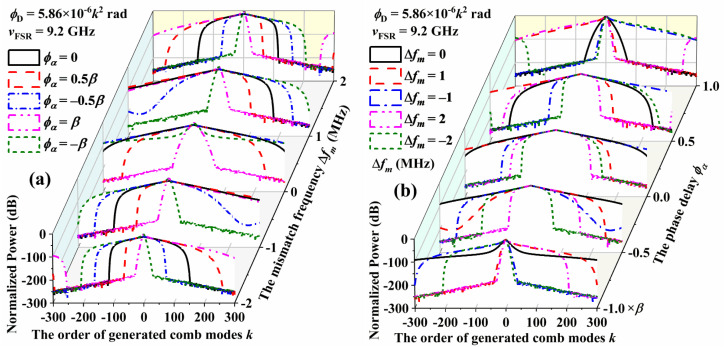
Simulated comb spectrum of the center ±300 combs with different mismatch phase delays *ϕ*_α_ and *ϕ*_Δ*f*_ with the constant dispersion phase delay *ϕ*_D_ of 5.86 × 10^−6^*k*^2^ rad. (**a**) The mismatch frequency Δ*f_m_* is set as the y-axis. (**b**) The mismatch phase delay *ϕ*_α_ is set as the y-axis.

A more comprehensive spectrum characterization should consider the variation of the mismatch phase delay *ϕ*_α_ and *ϕ*_Δ*f*_ with a constant dispersion phase delay *ϕ*_D_. By assuming the dispersion phase delay *ϕ*_D_ of 10 mm EOM being 5.86 × 10^−6^*k*^2^ rad, the EOM modulation frequency and the FSR of CEEOCG cavity both being 9.2 GHz, the simulation results of the comb spectrum are shown in Figure 7. In Figure 7a, the mismatch frequency Δ*f_m_* is set as the y-axis with scales of 0 MHz, ±1 MHz and ±2 MHz. The curves for different mismatch phase delays *ϕ*_α_ of 0, ±0.5*β* and ±*β* are presented with a black solid line (*ϕ*_α_ = 0), a red dash line (*ϕ*_α_ = 0.5*β*), a blue dash-dot line (*ϕ*_α_ = −0.5*β*), a magenta dash-dot-dot line (*ϕ*_α_ = *β*) and a green short dash line (*ϕ*_α_ = −*β*), respectively. Different from the analysis in Section 4.2, the mismatch phase delay *ϕ*_α_ is changed from ±0.8*β* to ±0.5*β* for a better separation of the curves in Figure 7. In each slice of the joint analysis in Figure 7a, the distribution of each comb spectrum corresponds to the variation of *ϕ*_α_ in sequence. For the slice of Δ*f_m_* = −2 MHz, the simulated curves are *ϕ*_α_ = −*β*, −0.5*β*, 0, 0.5*β*, and *β* successively from left to right. The CEEOCG comb spectrum is not symmetrical for a certain non-zero mismatch frequency Δ*f_m_*. For the mismatch frequencies Δ*f_m_* of the same absolute value but opposite sign, however, a horizontal inversion of the simulated CEEOCG comb spectrums can be found. With the increase of the absolute value of Δ*f_m_*, the power decay of higher order comb modes is significantly enhanced. When the normalized power is less than −180 dB, some jitters can be found. We attribute this phenomenon to the floating-point truncation error during the simulation. For the unexpected extra spectrum in the modes over the ±250-th order, the cause of formation is still not clear. More work will be conducted on this in the future.

In Figure 7b, the mismatch phase delay *ϕ*_α_ is set as the y-axis with scales of 0, ±0.5*β* and ±*β*. The curves for different mismatch frequencies Δ*f_m_* of 0 MHz, ±1 MHz and ±2 MHz are presented with a black solid line (Δ*f_m_* = 0 MHz), a red dash line (Δ*f_m_* = 1 MHz), a blue dash-dot line (Δ*f_m_* = −1 MHz), a magenta dash-dot-dot line (Δ*f_m_* = 2 MHz) and a green short dash line (Δ*f_m_* = −2 MHz), respectively. Due to the spectrum-narrowing effect of the mismatch frequency Δ*f_m_*, the distribution of CEEOCG spectrums in each slice of the same mismatch phase delay *ϕ*_α_ is not sequential with the increasing of Δ*f_m_*. However, the horizontal mirror effect of the simulated spectrums from opposite mismatch frequencies Δ*f_m_* is retained.

**Figure 8:**
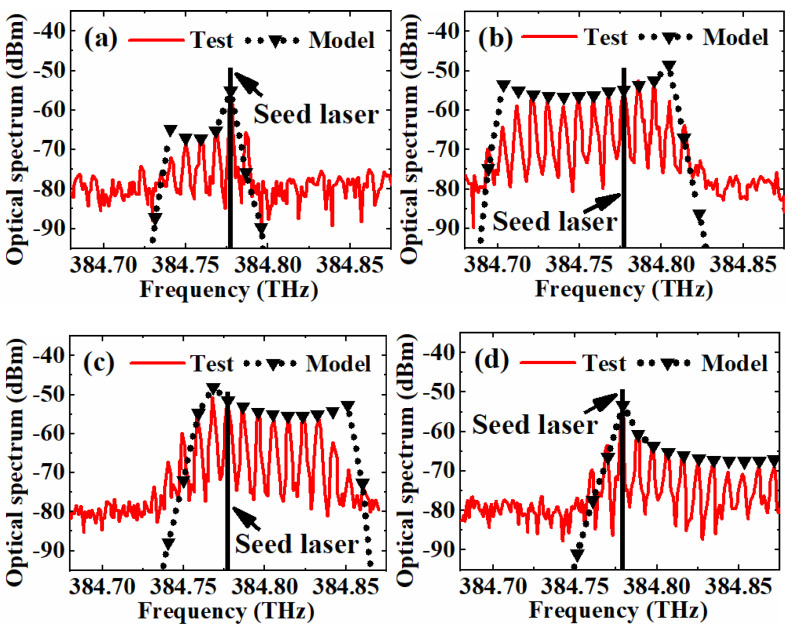
Curve fitting of four arbitrarily selected comb spectrums from an unstabilized CEEOCG with the proposed method. (**a**) *ϕ*_α_ = −0.98*β* and Δ*f_m_* = −110 MHz, (**b**) *ϕ*_α_ = 0.45*β* and Δ*f_m_* = 43 MHz, (**c**) *ϕ*_α_ = −0.7*β* and Δ*f_m_* = 50 MHz, (**d**) *ϕ*_α_ = *β* and Δ*f_m_* = −40 MHz.

The simulation results above are not only a verification of the proposed CEEOCG comb spectrum characterization method. They can be further applied to identify the working condition of CEEOCGs. In our previous work [37], the pre-adjustment of a CEEOCG is very time consuming in order to match the incident laser frequency, the cavity resonance and the EOM modulation frequency at the same time. It was quite a hard process as the generated comb is very unstable. One has to be very experienced to be able to identify the working condition of the CEEOCG. Then, the further fine tuning can be implemented properly. With the proposed method and the simulation results, it will not be a problem anymore. Based on the experimental setup established in Physikalisch-Technische Bundesanstalt [37], four CEEOCG comb spectrums were obtained and are shown in Figure 8. During the adjustment of the CEEOCG optical structure, these imperfect comb spectrums are present occasionally. With the dispersion phase delay *ϕ*_D_ calculated as 5.86 × 10^−6^*k*^2^ rad, the outer profile of the CEEOCG comb spectrums in Figure 8 is fitted with the simulation curves in Figure 7. The positive and negative polarity of the mismatch phase delay *ϕ*_α_ and frequency Δ*f_m_* can be first identified. Then, the very well-matched simulation curves can be obtained, with *ϕ*_α_ and Δ*f_m_* being −0.98*β* and −110 MHz for Figure 8a, 0.45*β* and 43 MHz for Figure 8b, −0.7*β* and 50 MHz for Figure 8c, *β* and −40 MHz for Figure 8d, respectively. By fine tuning the CEEOCG cavity length and the incident laser frequency according to the working condition identification above, the CEEOCG can be optimized to the ideal locking point in [37]. Therefore, the implementation of CEEOCG can be highly simplified with the help of the proposed comb spectrum characterization method.

## 5. Conclusions

In summary, we proposed an accurate and comprehensive comb spectrum characterization method for CEEOCGs. The optical electrical field with respect to the count of the round-trip propagation inside of CEEOCGs was accumulated. The content of the residual phase delay calculation equation in the proposed method ensures its applicability to the arbitrary working conditions of CEEOCGs. The simplification of the proposed method was accomplished without any mathematical approximation by using the Jacobi–Anger identity and Euler’s formula. With a maximum propagation count larger than 1000, the simulation error for the center ±300 comb modes comes from the truncation error of floating-point numbers only. Comparison results proved the error of the existing exponential approximation and the power coupling model to be linear and accelerated, increasing with the order of comb modes, respectively. Moreover, the proposed method can be efficiently computed with the Matrix multiplication and Hadamard product of matrices. By avoiding the iterative calculation of the power coupling among the generated comb modes, the simulation time can be reduced from 72.9 s down to 1.2 s.

To reveal the influence of the parameters of a CEEOCG, a series of simulations based on the proposed method were conducted with the key parameters of CEEOCGs independently and jointly. The independent introduction of different non-zero mismatch and dispersion phase delays all led to an obvious narrowing of the comb spectrums. With the increase of the comb mode order, however, the speed of the symmetrical power decay is different for each type of residual phase delay. More realistic simulations were conducted by analyzing the two types of mismatch phase delays jointly with a constant dispersion phase delay. Hence, the exact power of each comb mode can be predicted accurately and efficiently. To the best of our knowledge, this is the first comprehensive characterization of all the key parameters of CEEOCGs.

The simulations and analyses above were not only a verification of the proposed CEEOCG comb spectrum characterization method. They can be further applied to identify the working condition of CEEOCGs. Four arbitrarily selected comb spectrums from an unstabilized CEEOCG were fitted with the proposed method. A high consistency can be found between the test data and the simulated fitting results, further proving the applicability and accuracy of the proposed method. With the help of fitting parameters for such a working condition identification, the CEEOCG can be further fine-adjusted or even optimally redesigned. Accordingly, the CEEOCGs with either a spatial linear cavity or an integrated ring cavity can service the applications in optical communications and waveform synthesis with a better performance.

## Data Availability

Data underlying the results presented in this paper are not publicly available at this time but may be obtained from the authors upon reasonable request.

## Appendix A

In this appendix, the approximations during the derivation of the commonly applied exponential model is shown. This derivation based on the electrical field of the transmission beam from CEEOCG in Equation (3). According to the equivalent infinitesimal replacements of the exponent and sine items, exp(−*x*) and sin *y* are equivalent to 1 − *x* and *y*, respectively, when the variables *x* and *y* approach zero. To meet the condition for replacement in (3), the following analysis assumes that the residual phase delay *ϕ*_F_ and modulation phase delay *ω_m_t* both approaches to zero. In this case, the electrical field of the transmission beam from CEEOCG can be approximated as

(A1) $$E_{t}\approx E_{in}\frac{\left(1-R\right)\left(1-j\beta \omega_{m}t\right)}{1-R\left(1-j2\beta \omega_{m}t\right)}.$$

According to [43], Equation (A1) can be transformed to further analyze the electrical field of the transmitted comb as

(A2) $$E_{t}=E_{in}\frac{-\left(\frac{1-R}{2\beta R}\right)}{-\left(\frac{1-R}{2\beta R}\right)+j\omega_{m}t}+E_{in}\frac{j\left(\frac{1-R}{2\beta R}\right)\beta \omega_{m}t}{-\left(\frac{1-R}{2\beta R}\right)+j\omega_{m}t}.$$

If the power reflection coefficient *R* approaches 1, the first term of Equation (A2) *E_t_*_1_ can be approximated with a Fourier series as

(A3) $$E_{t1}=E_{in}\frac{-\left(\frac{1-R}{2\beta R}\right)}{-\left(\frac{1-R}{2\beta R}\right)+j\omega_{m}t}\approx E_{in}\sum_{k=0}^{\infty }\left(\frac{1-R}{2\beta R}\right)e^{-\left(\frac{1-R}{2\beta R}\right)k}e^{jk\omega_{m}t}.$$

Therefore, we can find the electrical field intensity of the *k*-th order sideband from the first term of Equation (A2) as

(A4) $$E_{tk1}=E_{in}\left(\frac{1-R}{2\beta R}\right)e^{-\left(\frac{1-R}{2\beta R}\right)k}.$$

At the same time, the factor *ω_m_t* in the second term of (A2) can be obtained with the following property of Fourier series.

(A5) $$\omega_{m}tg\left(\omega_{m}t\right)↔j\frac{dG_{k}\left(k\right)}{dk}.$$

Consequently, the electrical field intensity of the *k*-th order sideband from the second term of Equation (A2) *E_tk_*_2_ can be expressed as

(A6) $$E_{tk2}=E_{in}\beta {\left(\frac{1-R}{2\beta R}\right)}^{2}e^{-\left(\frac{1-R}{2\beta R}\right)k}.$$

As the power reflection coefficient *R* approaches 1 and the phase modulation index *β* is limited by the EOM to several rad, the intensity of the *k*-th order sideband in Equation (A6) is far smaller than the intensity in Equation (A4) and can be neglected. Considering the symmetrical power distribution of the positive and negative order of sidebands, the optical power intensity of the *k*-th order sideband can be finally approximated as

(A7) $$I_{tk}={\left|E_{in}\right|}^{2}{\left(\frac{1-R}{2\beta R}\right)}^{2}e^{-\left(\frac{1-R}{\beta R}\right)\left|k\right|}\approx {\left|E_{in}\right|}^{2}{\left(\frac{\pi }{2\beta F}\right)}^{2}e^{\frac{-\pi }{\beta F}\left|k\right|},$$

where *F* is the finesse of the CEEOCG cavity. For *R* approaches 1, *F* ≈ *πR*/(1 − *R*).
